# Engaging Spinal Networks to Mitigate Supraspinal Dysfunction After CP

**DOI:** 10.3389/fnins.2021.643463

**Published:** 2021-04-12

**Authors:** V. Reggie Edgerton, Susan Hastings, Parag N. Gad

**Affiliations:** ^1^Department of Neurobiology, University of California, Los Angeles, Los Angeles, CA, United States; ^2^Department of Neurosurgery, University of California, Los Angeles, Los Angeles, CA, United States; ^3^Brain Research Institute, University of California, Los Angeles, Los Angeles, CA, United States; ^4^Institut Guttmann, Hospital de Neurorehabilitació, Institut Universitari Adscrit a la Universitat Autònoma de Barcelona, Barcelona, Spain; ^5^SH Pediatric Physical Therapy, San Jose, CA, United States; ^6^Rancho Research Institute, Downey, CA, United States; ^7^SpineX, Inc., Los Angeles, CA, United States

**Keywords:** cerebral palsy, spinal cord, brain, muscle, EMG

## Abstract

Although children with cerebral palsy seem to have the neural networks necessary to generate most movements, they are markedly dysfunctional, largely attributable to abnormal patterns of muscle activation, often characterized as spasticity, largely reflecting a functionally abnormal spinal-supraspinal connectivity. While it is generally assumed that the etiologies of the disruptive functions associated with cerebral palsy can be attributed primarily to supraspinal networks, we propose that the more normal connectivity that persists between peripheral proprioception-cutaneous input to the spinal networks can be used to guide the reorganization of a more normal spinal-supraspinal connectivity. The level of plasticity necessary to achieve the required reorganization within and among different neural networks can be achieved with a combination of spinal neuromodulation and specific activity-dependent mechanisms. By engaging these two concepts, we hypothesize that bidirectional reorganization of proprioception-spinal cord-brain connectivity to higher levels of functionality can be achieved without invasive surgery.

## The Problem

It is generally assumed that the primary pathology of the nervous system that leads to cerebral palsy (CP) is located within and among different combinations of supraspinal networks and these pathologies can be due to multiple etiologies. In most cases, however, it appears that these supraspinally occurring pathologies also will be necessarily manifested as spinally mediated dysfunctions, affecting multiple peripheral sensory-motor systems involving equilibrium posture, locomotion, and trunk and head control (Smith and Gorassini, 2018). Although a high level of functionally immature connections are normally formed early in development, the pruning of neurons and synaptic connections occur subsequently using multiple, largely unknown, guidance mechanisms that result in the more effective connections between spinal networks and descending axons and proprioceptive afferents. But, if functionally immature or abnormal connections persist at the end of the early developmental phase, the supraspinal and propriospinal connectivity will result in abnormal sensory-motor responses. Persistence of these functionally abnormal synaptic connections are reinforced postnatally throughout the critical period of development and into adulthood, resulting in the commonly recognized neuromuscular disorders associated with CP, with the most common symptoms being collectively diagnosed as neuromuscular spasticity and stiffness of joints. More specifically, however, there is a persistent pervasiveness of poor coordination of the motor pools. Given the concept of coordination is so central to our basic hypothesis, it is important that the meaning of this concept be clearly defined. Often, a coordinated movement is one in which there is reciprocity of the dynamics of the temporal patterns of activation and deactivation of flexor and extensor motor pools that generate the movement. “Normal” movements, however, are generated with a continuum of differing degrees of overlap and changes in levels of activation and deactivation. It is useful to be aware that the motor task that is being generated is defined largely by the temporal patterns of activation of motor pools. For example, many of the same muscles are activated when stepping forward, backward and sideways, but the patterns of muscles activated differ substantially (Shah et al., 2012). The degree of reciprocity vs. co-activation varies considerably among the uninjured general population. In individuals with symptoms of spasticity, there is an unusually greater coincidence of co-contractions of flexor and extensor motor pools movements that would be considered poorly coordinated. Theoretically, the greater the number of connections that develop between the brain and spinal cord that are functionally aberrant, the fewer normal targets that remain accessible (Bennett et al., 1983; Callaway et al., 1989; Alexeeva et al., 1997; Maegele et al., 2002). A fundamental and essential driver in the biological design of our nervous systems, phylogenetically, ontogenetically and epigenetically in reaching the normal targets undoubtedly has been earth’s gravitational vectors (Edgerton et al., 2000). We reason that this fundamental feature in the strategy of our sensory-motor design highlighted here is that the difficulty in accommodating gravity is consistently and pervasively revealed in individuals with CP, as children and in adults. So many of their sensory-motor challenges are linked to maintaining equilibrium while moving effectively in a 1G environment (Recktenwald et al., 1999). It is clearly evident in the motor behaviors of individuals with CP that the neural connectivity did not develop appropriately for this uniformly present fundamental gravitational challenge. For the reasons noted above our interventional strategy as presented has been focused functionally on the necessity of realigning the sensory-motor connectivity to accommodate to normal gravitational vectors. Theoretically, to regain a normal supraspinal-spinal connectivity to earth’s gravitational forces, the earlier maladaptive state that was learned postnatally to sustain equilibrium while in an abnormal state, must be re-transformed to achieve a normal translation of sensory input in a 1G environment (Smith and Gorassini, 2018; Cappellini et al., 2020).

## Solution

Given our experience with spinal cord injury (Gerasimenko Y. et al., 2015; Reggie et al., 2018), which has similar, and in some ways more severe functional aberrations than in CP, we developed interventions designed to transform functionally aberrant brain-spinal connections to a greater prominence of functionally normal connections. We reasoned that we could do this by maximizing the dominance of proprioception and the spinal networks that translate this sensory input and minimizing the pathology of the brain, in controlling posture and locomotion. Cappellini et al. (2016, 2020) noted how much the dysfunction of gait in children with CP can be related to spinal neuronal networks vs. supraspinal dysfunction. A more thorough knowledge about pattern generation circuitries in infancy may improve our understanding of developmental motor disorders, highlighting the necessity for regulating the functional properties of abnormally developed neuronal locomotor networks as a target for early sensorimotor rehabilitation. Similarly a very tight link was described between the activity patterns of populations of pyramidal tract neurons and the biomechanics of unconstrained locomotion in cats (Prilutsky et al., 2005). Versteeg et al. (2021) reported that cuneate nucleus neurons have muscle-like properties that have a greater sensitivity to active than passive movements of the upper limb and that their receptive fields resemble single muscles. These observations suggest that muscle specific signals proprioceptive input could have an activity-dependent impact on supraspinal networks that could transform dysfunctional neural networks to a more functional state. There is extensive evidence that the neural networks of an individual with CP can learn more effective movement skills as it does after spinal injury (Dewar et al., 2015; Morgan et al., 2015; Reid et al., 2015). Thus, we hypothesize that spinal neuromodulation in concert with proprioceptive-driven activity-dependent mechanisms of spinal networks can transform the supraspinal-spinal dysfunctional connectivity of CP into highly functional connections, improving the functionality of networks. The result should be the recovery of motor tasks that are more forceful, powerful, efficient and display finer control as needed in a 1G environment. This hypothesis is counter to the predominant thinking that all of the “motor” functions, noted above are controlled largely by the brain, rather than the spinal cord. Ironically, proprioception is considered to be a major contributor to the spasticity in CP, and, therefore, is a primary target to reduce the tonic stiffness by performing selective dorsal root rhizotomy (Mortenson et al., 2021). However, selective rhizotomy minimizes the sensor input, which normally plays a prominent role in controlling movements and can disrupt autonomic functions.

Multiple clues led us to the logic applying a specialized neuromodulatory technique in combination with subject specific activity-dependent rehabilitation. First, our studies, and that of many other labs for decades clearly tell us that proprioception plays a more important role than occasionally correcting mistakes, as in tripping, when there is not enough time to adjust the planned movements (Gerasimenko et al., 2017). Proprioception plays a central role in the details of controlling posture, locomotion, and fine motor tasks, even when there is no connectivity between the spinal networks and the brain (Grillner and Zangger, 1975; de Leon et al., 1999). Some specific observations suggesting a possible crucial role for spinal networks in individuals with CP to facilitate functional recovery are: (1) After a complete, mid-thoracic spinal transection a mammal can step forward, backward or sideways and at speeds appropriate to the speed of the treadmill belt. (2) Humans that have lost proprioception as an adult are essentially, functionally paralyzed, at least for months, even though all descending motor pathways remain intact. (3) Given that spinal networks can readily learn to perform a new motor task without help from the brain, we reasoned that the potential level of plasticity among spinal and supraspinal networks in the presence of spinal neuromodulation combined with skilled therapy can be robust enough to supplant the original supraspinal disruptive connectivity and reinforce more normal functional connections with a greater presence of normal sensory input associated with routine motor tasks via activity-dependent mechanisms. (4) Another clue, suggests that much of the aberrant connectivity is inextricably linked to skills associated with equilibrium, i.e., with gravity being of a constant presence in the evolution of all life on Earth (Wallard et al., 2014, 2018). It seems obvious that the network connectivity design of sensory motor functions that has evolved phylogenetically, has specifically accommodated to earth’s gravity, and that there are numerous motor dysfunctions that reflect that this feature was not carried out normally, either ontogenetically or epigenetically in many CP cases. And finally, and (5) could the fact that CP being a developmental phenomenon be an advantage in using interventional strategies to take advantage of higher levels of neuroplasticity during the critical period of development of motor primitives as suggested by Bizzi and colleagues (Overduin et al., 2015) and hypothesized by Edelman (1993) as Neural Darwinism and Neuronal Group Selection, presumably of the brain, and Edgerton and colleagues as a similar phenomenon in the spinal cord (Edgerton et al., 2001).

## Testing the Hypothesis

While the biological bases of the underlying concepts may seem sound, there has not, however, been a single systems-level concept relevant to the etiology of CP as we have proposed. Technically it is now feasible to induce systems level changes in specific functional connectivity of networks patterns of sensory input that translates to a predictable motor output by repetitively activating specific proprioception patterns as occurs during stand training that enables standing ability but not stepping ability (de Leon et al., 1999), facilitates changes at the level of synapses and neural networks and changes the expression of genes that could mediate the synaptic adaptations to locomotor training after a spinal hemisection. More comprehensive, quantitative assays under clinically controlled conditions on a limited population of subjects could be performed with present-day technologies. Clear evidence of the validity of the proposed mechanisms at a systems level of understanding seems to be not only feasible with proper resources, but in addition a case can be made for the urgency of such an effort based on it’s potential efficacy and safety as demonstrated from more than a decade of studies of human subjects with a similar non-invasive interventional strategy in recovering functions after SCI (Gerasimenko Y. P. et al., 2015; Rath et al., 2018; Sayenko et al., 2018; Gad et al., 2020; Kreydin et al., 2020).

**FIGURE 1:**
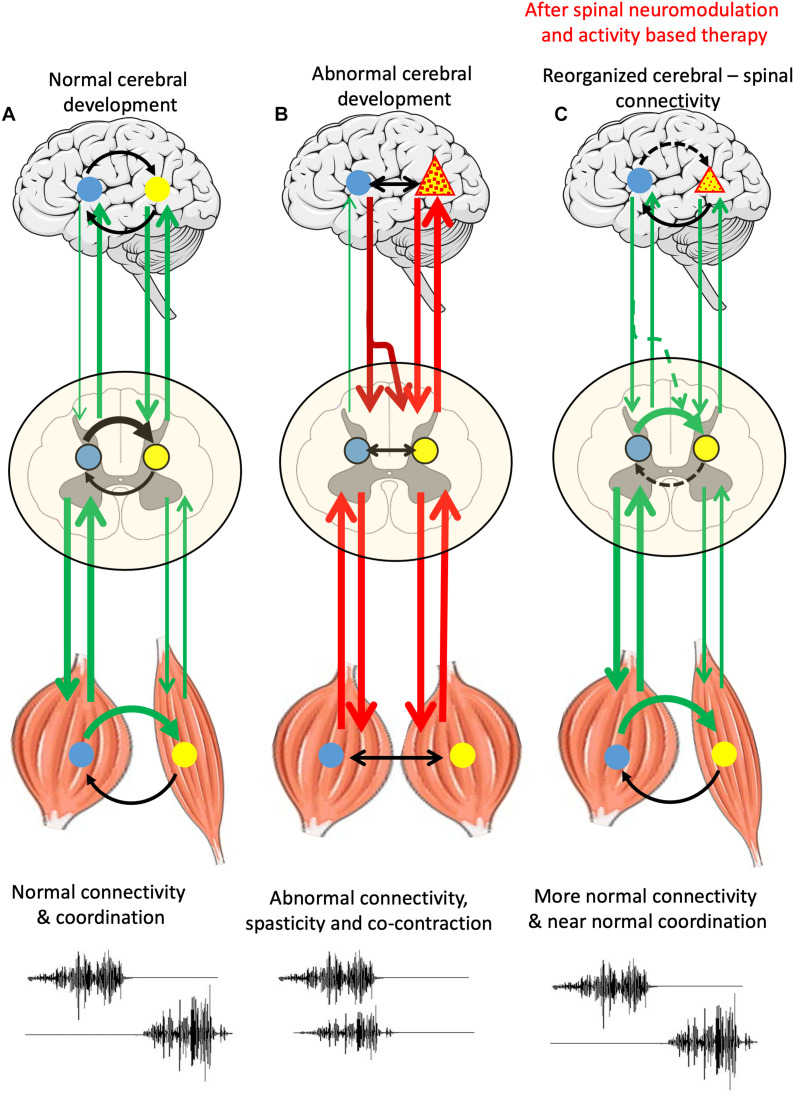
Schematic representation of supraspinal-spinal-muscle connectivity in normal cerebral development, abnormal cerebral development and in abnormal cerebral development after spinal neuromodulation and activity-based therapy **(A)** normal brain-spinal networks and muscles with sensors, bidirectionally communicating input and output signals forming a complete loop, including reciprocal EMG of agonist and antagonist muscles, see two channels at the bottom. **(B)** A region of supraspinal pathology (triangle) resulting in aberrant descending signals causing disruptive degrees of co-contractions of flexor and extensor motor pools and muscles. **(C)** A remodeling process can of supraspinal and spinal networks can begin with a combination of a non-invasive electrical neuromodulation technique that empowers the spinal networks to begin to assume a dominating control of normalizing the coordination of flexor and extensor motor pools. With repetitive practice in the presence of neuromodulation, we propose that there will be significant reorganization toward a gradually occurring normalization of supraspinal and spinal networks.

Figure 1 illustrates the experimental outcomes that we hypothesize can be obtained with a patient with CP. We hypothesize that applying a combination of non-invasive spinal neuromodulation and specific activity-dependent mechanisms to an individual with CP can trigger a bidirectional reorganization of proprioception-spinal cord-brain connectivity to a functional state that can generate near normal postural and locomotor functions. An individual free of abnormalities are continuously signaling sensory-motor information bi-directionally between the brain and spinal cord and the kinetic and kinematic sensors (proprioception) in muscle, tendons, and skin (Figure 1A). CP is generally considered to be a cerebral pathology (Figure 1B) which will send abnormal motor signals due to the spinal networks which in turn will project abnormal signals to predominately flexor or extensor motor pools and muscles. As opposed to the spinal networks generating a highly coordinated agonist-antagonist pattern (Figure 1A), the disruptive, aberrant, descending commands from the brain generates abnormal levels of co-contraction signals among the spinal interneuronal networks that induces high levels of co-contractions of flexors and extensor muscles. The first key point here is that the disruptive signals from the brain, then spinal, then muscular, results in abnormal proprioceptive signal to the spinal networks which we now know sends muscle specific signals to multiple supraspinal nuclei. Thus, basically a continuous loop of abnormal signals imposes persistent abnormal sensory-motor signals that results in a reorganization of networks that reflect the initial supraspinal pathology. Fortunately, there is reason to predict that there are several key points that can be used to interrupt the abnormal sensory-motor loop to a much greater level of normality. These points are: (1) The disruptive spinal networks can be transformed to a more normal physiological state with newly developed electrical neuromodulation combined with activity-dependent modulation via a near normal pattern of proprioceptive ensembles project to the spinal networks. (2) Proprioceptive input has the potential to overcome the disruptive descending input and reorganize the connectivity into a state that generates coordinated movements. And, (3) There is strong evidence that a similar normalizing process can occur in the more normal ascending input to supraspinal centers can then form more normal networks in the descending motor signals. Experiments in which CP patients attempt to perform motor task in the presence and absence of neuromodulation while recording supraspinal and spinally evoked potentials and recording EMG activities of different combinations of muscles and kinematics and kinetics to characterize the motor task will provide the data that can determine whether the proposed hypothesis should be accepted, at least from a functional, systems level.

## Disclosure

VE holds shareholder interest in NeuroRecovery Technologies and hold certain inventorship rights on intellectual property licensed by the Regents of the University of California to NeuroRecovery Technologies and its subsidiaries. VE and PG holds shareholder interest in SpineX, Inc. and hold certain inventorship rights on intellectual property licensed by the Regents of the University of California to SpineX, Inc.

## Data Availability Statement

The original contributions presented in the study are included in the article/supplementary material, further inquiries can be directed to the corresponding author.

## Author Contributions

All authors listed have made a substantial, direct and intellectual contribution to the work, and approved it for publication.

## Conflict of Interest

PG was employed by SpineX. SH was employed by Susan Hastings Pediatric Clinic. The remaining author declares that the research was conducted in the absence of any commercial or financial relationships that could be construed as a potential conflict of interest.

## References

[B1] Alexeeva N. Broton J. G. Suys S. Calancie B. (1997). Central cord syndrome of cervical spinal cord injury: widespread changes in muscle recruitment studied by voluntary contractions and transcranial magnetic stimulation. *Exp. Neurol.* 148 399–406. 10.1006/exnr.1997.6689 9417819

[B2] Bennett M. R. McGrath P. A. Davey D. F. Hutchinson I. (1983). Death of motorneurons during the postnatal loss of polyneuronal innervation of rat muscles. *J. Comp. Neurol.* 218 351–363. 10.1002/cne.902180311 6886080

[B3] Callaway E. M. Soha J. M. Van Essen D. C. (1989). Differential loss of neuromuscular connections according to activity level and spinal position of neonatal rabbit soleus motor neurons. *J. Neurosci.* 9 1806–1824. 10.1523/jneurosci.09-05-01806.1989 2723750 PMC6569826

[B4] Cappellini G. Ivanenko Y. P. Martino G. MacLellan M. J. Sacco A. Morelli D. (2016). Immature Spinal Locomotor Output in Children with Cerebral Palsy. *Front. Physiol.* 7:478. 10.3389/fphys.2016.00478 27826251 PMC5078720

[B5] Cappellini G. Sylos-Labini F. Dewolf A. H. Solopova I. A. Morelli D. Lacquaniti F. (2020). Maturation of the Locomotor Circuitry in Children With Cerebral Palsy. *Front. Bioeng. Biotechnol.* 8:998. 10.3389/fbioe.2020.00998 32974319 PMC7462003

[B6] de Leon R. D. Tamaki H. Hodgson J. A. Roy R. R. Edgerton V. R. (1999). Hindlimb locomotor and postural training modulates glycinergic inhibition in the spinal cord of the adult spinal cat. *J. Neurophysiol.* 82 359–369. 10.1152/jn.1999.82.1.359 10400964

[B7] Dewar R. Love S. Johnston L. M. (2015). Exercise interventions improve postural control in children with cerebral palsy: a systematic review. *Dev. Med. Child Neurol.* 57 504–520. 10.1111/dmcn.12660 25523410

[B8] Edelman G. M. (1993). Neural Darwinism: selection and reentrant signaling in higher brain function. *Neuron* 10 115–125. 10.1016/0896-6273(93)90304-a 8094962

[B9] Edgerton V. R. Roy R. R. De Leon R. D (2001). “Neural Darwinism in the mammalian spinal cord,” in *Spinal Cord Plasticity: Alterations in Reflex Function*, eds Patterson M. M. Graw J. W. (Boston: Kluwer Academic), 185–206. 10.1007/978-1-4615-1437-4_8

[B10] Edgerton V. R. Roy R. R. Hodgson J. A. Day M. K. Weiss J. Harkema S. J. (2000). How the science and engineering of spaceflight contribute to understanding the plasticity of spinal cord injury. *Acta Astronaut.* 47 51–62. 10.1016/s0094-5765(00)00009-6 11543389

[B11] Gad P. Kreydin E. Zhong H. Edgerton V. R. (2020). Enabling respiratory control after severe chronic tetraplegia: an exploratory case study. *J. Neurophysiol.* 124 774–780. 10.1152/jn.00320.2020 32755339 PMC7509292

[B12] Gerasimenko Y. Gorodnichev R. Moshonkina T. Sayenko D. Gad P. Reggie Edgerton V. (2015). Transcutaneous electrical spinal-cord stimulation in humans. *Ann. Phys. Rehabil. Med.* 58 225–231. 10.1016/j.rehab.2015.05.003 26205686 PMC5021439

[B13] Gerasimenko Y. P. Lu D. C. Modaber M. Zdunowski S. Gad P. Sayenko D. G. (2015). Noninvasive Reactivation of Motor Descending Control after Paralysis. *J. Neurotrauma* 32 1968–1980. 10.1089/neu.2015.4008 26077679 PMC4677519

[B14] Gerasimenko Y. Sayenko D. Gad P. Liu C. T. Tillakaratne N. J. K. Roy R. R. (2017). Feed-Forwardness of Spinal Networks in Posture and Locomotion. *Neuroscientist* 23 441–453. 10.1177/1073858416683681 28403746 PMC5495622

[B15] Grillner S. Zangger P. (1975). How detailed is the central pattern generation for locomotion? *Brain Res.* 88 367–371. 10.1016/0006-8993(75)90401-1 1148835

[B16] Kreydin E. Zhong H. Latack K. Ye S. Edgerton V. R. Gad P. (2020). Transcutaneous Electrical Spinal Cord Neuromodulator (TESCoN) Improves Symptoms of Overactive Bladder. *Front. Syst. Neurosci.* 14:1. 10.3389/fnsys.2020.00001 32116576 PMC7017715

[B17] Maegele M. Muller S. Wernig A. Edgerton V. R. Harkema S. J. (2002). Recruitment of spinal motor pools during voluntary movements versus stepping after human spinal cord injury. *J. Neurotrauma* 19 1217–1229. 10.1089/08977150260338010 12427330

[B18] Morgan C. Novak I. Dale R. C. Badawi N. (2015). Optimising motor learning in infants at high risk of cerebral palsy: a pilot study. *BMC Pediatr.* 15:30. 10.1186/s12887-015-0347-2 25880227 PMC4389951

[B19] Mortenson P. Sadashiva N. Tamber M. S. Steinbok P. (2021). Long-term upper extremity performance in children with cerebral palsy following selective dorsal rhizotomy. *Childs Nerv. Syst.* 10.1007/s00381-020-05018-2 [Epub ahead of print]. 33386960

[B20] Overduin S. A. d’Avella A. Roh J. Carmena J. M. Bizzi E. (2015). Representation of Muscle Synergies in the Primate Brain. *J. Neurosci.* 35 12615–12624. 10.1523/jneurosci.4302-14.2015 26377453 PMC4571600

[B21] Prilutsky B. I. Sirota M. G. Gregor R. J. Beloozerova I. N. (2005). Quantification of motor cortex activity and full-body biomechanics during unconstrained locomotion. *J. Neurophysiol.* 94 2959–2969. 10.1152/jn.00704.2004 15888524

[B22] Rath M. Vette A. H. Ramasubramaniam S. Li K. Burdick J. Edgerton V. R. (2018). Trunk Stability Enabled by Noninvasive Spinal Electrical Stimulation after Spinal Cord Injury. *J. Nneurotrauma* 35 2540–2553. 10.1089/neu.2017.5584 29786465 PMC6205803

[B23] Recktenwald M. R. Hodgson J. A. Roy R. R. Riazanski S. McCall G. E. Kozlovskaya I. (1999). Effects of spaceflight on rhesus quadrupedal locomotion after return to 1G. *J. Neurophysiol.* 81 2451–2463. 10.1152/jn.1999.81.5.2451 10322080

[B24] Reggie E. V. Yury G. Parag G. Dimitry S. (2018). Basic concepts underlying activity-dependent mechanisms in the rehabilitation of sensory-motor function after spinal cord injury. *Spinal Cord Med.* 54 897–911.

[B25] Reid L. B. Rose S. E. Boyd R. N. (2015). Rehabilitation and neuroplasticity in children with unilateral cerebral palsy. *Nat. Rev. Neurol.* 11 390–400. 10.1038/nrneurol.2015.97 26077839

[B26] Sayenko D. G. Rath M. Ferguson A. R. Burdick J. Havton L. A. Edgerton V. R. (2018). Self-assisted standing enabled by non-invasive spinal stimulation after spinal cord injury. *J. Neurotrauma.* 36 1435–1450. 10.1089/neu.2018.5956 30362876 PMC6482915

[B27] Shah P. K. Gerasimenko Y. Shyu A. Lavrov I. Zhong H. Roy R. R. (2012). Variability in step training enhances locomotor recovery after a spinal cord injury. *Eur. J. Neurosci.* 36 2054–2062. 10.1111/j.1460-9568.2012.08106.x 22591277 PMC3389255

[B28] Smith A. T. Gorassini M. A. (2018). Hyperexcitability of brain stem pathways in cerebral palsy. *J. Neurophysiol.* 120 1428–1437. 10.1152/jn.00185.2018 29947590

[B29] Versteeg C. Chowdhury R. H. Miller L. E. (2021). Cuneate nucleus: The somatosensory gateway to the brain. *Curr. Opin. Physiol.* 20 206–215. 10.1016/j.cophys.2021.02.004 33869911 PMC8049169

[B30] Wallard L. Dietrich G. Kerlirzin Y. Bredin J. (2014). Balance control in gait children with cerebral palsy. *Gait Posture* 40 43–47. 10.1016/j.gaitpost.2014.02.009 24656683

[B31] Wallard L. Dietrich G. Kerlirzin Y. Bredin J. (2018). Effect of robotic-assisted gait rehabilitation on dynamic equilibrium control in the gait of children with cerebral palsy. *Gait Posture* 60 55–60. 10.1016/j.gaitpost.2017.11.007 29156378

